# The Prognostic Effect of Multidisciplinary Team Intervention in Patients with Advanced Gastric Cancer

**DOI:** 10.3390/curroncol29020102

**Published:** 2022-02-17

**Authors:** Yuan-Yuan Xiang, Cun-Can Deng, Han-Yuan Liu, Zi-Chong Kuo, Chang-Hua Zhang, Yu-Long He

**Affiliations:** 1 Digestive Disease Center, Seventh Affiliated Hospital of Sun Yat-Sen University, Shenzhen 518107, China; xiangyy8@mail2.sysu.edu.cn (Y.-Y.X.); dengcc@mail2.sysu.edu.cn (C.-C.D.); liuhy97@mail2.sysu.edu.cn (H.-Y.L.); kuozc@mail2.sysu.edu.cn (Z.-C.K.); 2 Department of Gastrointestinal Surgery, First Affiliated Hospital of Sun Yat-Sen University, Guangzhou 510080, China

**Keywords:** gastric cancer (GC), multidisciplinary team (MDT), prognosis

## Abstract

Background: The effect of multidisciplinary team intervention (MDT) on the prognosis of advanced gastric cancer (GC) is still controversial. This study aims to analyze the effect of MDTs on the overall survival time of advanced gastric cancer patients. Methods: Patients with advanced GC who underwent surgical treatment between 2007 and 2014 were included in the study. They were divided into two groups; the MDT group received MDT treatment and the non-MDT group received conventional treatment. The Kaplan-Meier method was used to compare the overall survival (OS) of the two groups. The prognostic factors of advanced GC were evaluated by multivariate Cox regression analysis. Results: 394 patients were included in our study. Kaplan-Meier survival analysis showed that the prognosis of advanced GC patients with who underwent MDT intervention was better than those without (3-year OS of 55.6% vs. 46.1%, *p* = 0.005), Multivariate analysis indicated that MDT intervention could reduce mortality (HR = 0.493, *p* < 0.001). Conclusions: MDT intervention is an effective measure that improves the survival of patients with advanced GC.

## 1. Introduction

According to global cancer data in 2018, gastric cancer (GC) is ranked as the fifth most common malignant tumor worldwide and consequently, the third common cause of cancer-related deaths. Japan and Korea have a higher incidence among the East Asian countries, while North America and North Africa observed a lower morbidity rate [1]. Modern medical diagnosis and treatment technology have improved rapidly over the years, bringing about vast leaps in the screening, diagnosis and treatment of GC. While there are some accurate and effective treatment plans for early GC, advanced GC patients are not as lucky, leaving them stranded with no clear or universal treatment plan [2,3,4]. A multidisciplinary team (MDT) is composed of experts from various medical departments who are engaged in the diagnosis and treatment process to ensure that the patient is provided with the best diagnosis and treatment plan. An effective MDT can improve the diagnosis rate of early GC patients and prolong their overall survival time [5,6]. For advanced GC, due to the complexity usually associated with patients with this disease, doctors often make critical decisions based solely on past experience and related clinical guidelines. Therefore, MDTs were envisioned to be the solution that will potentially untangle the myriad of problems involved [7]. Some studies have found that an MDT discussion could improve the accuracy of malignant tumor staging and provide patients with better personalized treatment decisions [6,8]. The MDT team generally discusses the diagnosis and treatment of complex diseases such as cancer on a regular basis (in our facility, weekly), and formulates a detailed diagnosis and treatment plan in accordance with current guidelines [9,10].

According to the National Comprehensive Cancer Network (NCCN) Guidelines for Gastric Cancer 2013, the diagnosis and treatment of gastric cancer should be managed by an MDT [11]. This way, GC patients will benefit for many reasons. For one, MDT can reduce unnecessary tumor staging tests and shorten the time to initiate treatment. The relevant departments involved are capable of evaluating the condition, determining the tumor stage, formulating diagnosis and treatment plans, and thus providing personalized treatment in time. This comprehensive management of patients is proven to improve the efficiency of diagnosis and treatment of gastric cancer [12]. Secondly, part of the diagnosis of patients managed by MDTs would improve [13,14], with their treatment plans revised for the better [15] after MDT discussions, due to a more accurate and complete preoperative staging result, and suitable neoadjuvant and adjuvant treatment. However, there is little solid evidence that MDT can prolong the survival time of patients, albeit controversially [13]. MDTs can change the treatment plan of even the seasoned clinicians. Some research results showed that about 23.0–41.7% of patients’ treatment plans were altered after an MDT discussion, mainly due to the change in initial diagnosis or pathological staging [15,16]. Finally, MDT can increase the overall survival time and prognosis of early GC patients [5].

However, there is still a lack of strong clinical studies on the therapeutic impact of MDT in advanced GC. A few studies had shown that MDT therapy improved prognosis in patients with advanced GC [17,18]. The prognosis effect in advanced GC is still inconclusive. Thus, we had collected the clinical information of advanced GC patients who were provided MDT treatment and non-MDT treatment, and further studied whether MDT intervention can improve the survival of advanced GC.

## 2. Materials and Methods

### 2.1. Basic Characteristics of Patients

We had gathered clinicopathological and follow-up data of patients first diagnosed with stage III and stage IV GC in the same hospital from January 2007 to December 2014. Inclusion criteria: (1) gastric cancer diagnosed by pathology; (2) clinical stage III/IV according to AJCC (the American Joint Committee on Cancer) 8th staging system; (3) all patients received surgery and postoperative adjuvant therapy, and the surgery was performed by doctors in the same medical group. All patients received perioperative and operative treatment. Exclusion criteria: inoperable extensive metastases or multiple malignancies, emergency surgery, incomplete follow-up data. There were two groups in our study: the MDT group who received MDT intervention, and the non-MDT group who received treatment based on the clinicians’ experience. Patients’ clinical information included age, sex, tumor size, location, depth of invasion, lymph node metastasis, tumor grade, Borrmann’s classification, tumor stage (according to AJCC 8th edition), and carcinoembryonic antigen (CEA). The study was approved by the medical ethics committee of the seventh Affiliated Hospital of Sun Yat-sen University on 18 March 2021 (No.: KY-2020-024-01). This study is consistent with the Declaration of Helsinki.

### 2.2. MDT Intervention

Since 2012, we have held weekly discussions with the MDT board. All patients with advanced GC were treated via an MDT. All patients with pathological diagnosis of stage III or IV advanced GC should undergo MDT discussion. Prior to the discussion, we informed the patients that their condition required MDT attention. The discussion was generally led by the attending doctor, and related departments were invited. The MDT usually includes radiologists, gastrointestinal surgeons, oncologists, radiotherapists, anesthesiologists, thoracic surgeons, hepatobiliary surgeons, pathologists, nutritionists and specialist nurses. The focus is placed on tumor staging and a subsequent treatment plan, including preoperative neoadjuvant chemotherapy, surgery, postoperative adjuvant therapy, etc. After the discussion, a detailed and specific treatment plan would be formed. The attending doctor will then inform the patients and their families of the treatment plan. Once agreed upon, he will then supervise its execution and adherence.

### 2.3. Patients’ Follow-Up Data

After treatment, patients were reassessed trimonthly for the first year, twice a year for the following 4 years, and yearly thereafter. They received regular enhanced chest and abdomen CT, endoscopy, and serum tumor marker tests. Follow-up data were available until December 2017.

### 2.4. Data Analysis

Quantitative data were analyzed using *t*-test, while categorical variables were handled with chi-square test and Fisher’s exact test, and were described in percentage. the overall survival rates between the two groups were compared using Kaplan-Meier analysis and log-rank tests. In accordance with the proportional hazard (PH) assumption, the variables were included in the univariate analysis. Multivariate analysis was selected to investigate multiple variables and the enter method was used to determine independent prognostic factors. *p* < 0.05 was considered statistically significant. Statistical analyses were preformed using GraphPad Prism 8.0 (GraphPad, San Diego, CA, USA).

## 3. Results

### 3.1. Clinical Characteristics of Patients

**Figure 1:**
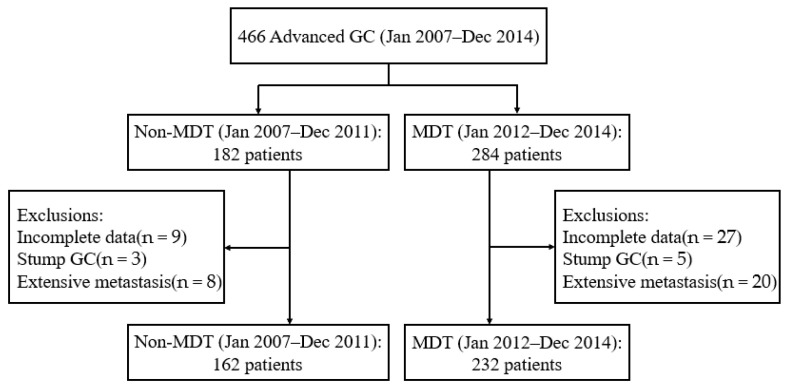
Patient flow chart description.

According to the research criteria, a total of 394 cases of advanced GC were included. Of those, 232 and 162 patients with advanced GC were placed in the MDT and non-MDT group, respectively (Figure 1). A total of six patients were lost in follow-up due to loss of contact, and thus the rate of follow up was 98.5%. Then, the mean follow-up time was 36.78 ± 22.80 months, while the median follow-up time was 33.50 months. Due to the lower number of T1 and T2 cases, they were combined into the ‘T1 + T2’ stage. The ‘moderately differentiated’ and ‘poorly differentiated’ classes were classified according to the proportion of differentiation. Between the two groups, no significant differences were found in the following aspects: age, sex, primary tumor site, tumor grade, Borrmann classification, TNM stage, CEA level and radical resection rate (Table 1). The percentage of T4 stage in the MDT and non-MDT groups was 59.1% and 32.1%, respectively (*p* < 0.001), while the percentage of N3 stage was 45.3% in the MDT group and 30.9% for the non-MDT group (*p* < 0.001).

### 3.2. The Prognosis Effect of MDTs in Advanced GC

**Figure 2:**
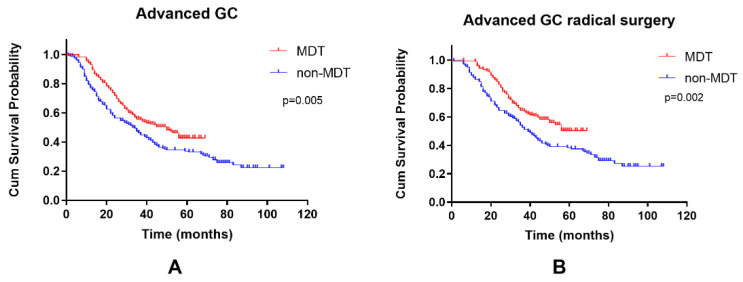
Kaplan-Meier curves of the OS of the two groups. (**A**) Advanced GC (**B**) underwent radical surgery of advanced GC.

The OS (overall survival) rates were calculated by the Kaplan-Meier method. The survival curves of the two groups were compared by log-rank test. The overall survival rate of the MDT group was higher than that of the non-MDT group (3-year OS of 55.6% vs. 46.1%, *p* = 0.005) (Figure 2A). The OS rate of the MDT group was higher than those of the non-MDT group after radical surgery as well (3-year OS of 64.6% vs 54.2%, *p* = 0.002) (Figure 2B). Multivariate analysis showed that MDT intervention and radical surgery were protective factors that decreased the mortality of advanced GC (HR = 0.493, *p* < 0.001; HR = 0.127, *p* < 0.001), while stomach body carcinoma, poorly differentiated adenocarcinoma, N3 stage and elevated CEA were adverse factors that increases the mortality in advanced GC (Table 2).

To further investigate the role of MDT intervention in specific staging, we individually studied the impact of MDT on the prognosis of different stages.

### 3.3. Clinical Characteristics of Stage III Gastric Cancer Patients

There were 294 cases of stage III GC, with 122 cases in the non-MDT group, and 172 cases in the MDT group. No statistical differences were found in age, sex, tumor size, location, degree of differentiation, Borrmann classification, CEA level or radical resection rate. In terms of invasiveness, the percentage of stage T4 in the MDT group was 55.2% and the rate of stage T4 in the non-MDT group was 32.0% (*p* < 0.001); the rate of stage N3 was 39.0% in the MDT group and the rate of stage N3 was 26.2% in the non-MDT group (*p* < 0.001) (Supplementary Table S1).

### 3.4. The Prognostic Effect of MDTs in Stage III Gastric Cancer

**Figure 3:**
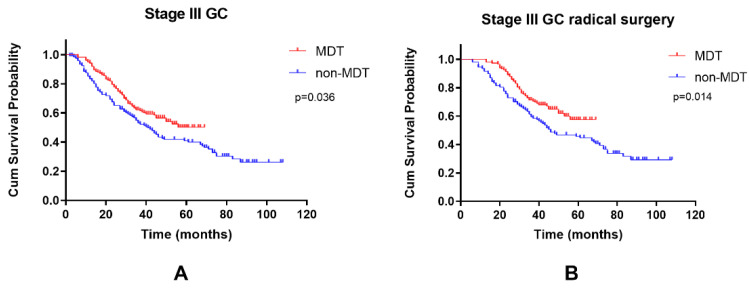
Kaplan-Meier curves of the OS of the two groups. (**A**) Stage III GC (**B**) stage III GC which underwent radical surgery.

Among stage III GC patients, the 3-year OS rate of the MDT group was 62.1%, while that of the non-MDT group was calculated to be 54.8% (*p* = 0.036) (Figure 3A). The 3-year overall survival rate of MDT patients with stage III GC who underwent radical surgery was 71.2%, compared with 59.5% in the non-MDT group (*p* = 0.014) (Figure 3B). Multivariate analysis showed that MDT intervention and radical surgery were protective factors that reduces the mortality of stage III GC (HR = 0.504, *p* < 0.001; HR = 0.044, *p* < 0.001), while gastric body carcinoma, poorly differentiated adenocarcinoma, N3 stage and elevated CEA were adverse factors that increased the mortality of advanced GC (Table 3).

### 3.5. Clinical Characteristics of Stage IV Gastric Cancer Patients

There were 100 patients diagnosed with stage IV GC, with 40 cases in the non-MDT group and 60 cases in the MDT group. There were no statistical differences between the two groups in terms of age, sex, tumor size, tumor location, tumor grade, Borrmann classification, CEA level or radical resection rate. The proportion of stage T4 in MDT patients with stage IV GC was higher than that in non-MDT patients (70.0% vs. 32.5%), compared with the non-MDT group, the proportion of stage T3 in the MDT group was lower. (28.3% vs. 62.5%, *p* = 0.001) (Supplementary Table S2).

### 3.6. The Prognostic Effect of MDTs in Stage IV GC

**Figure 4:**
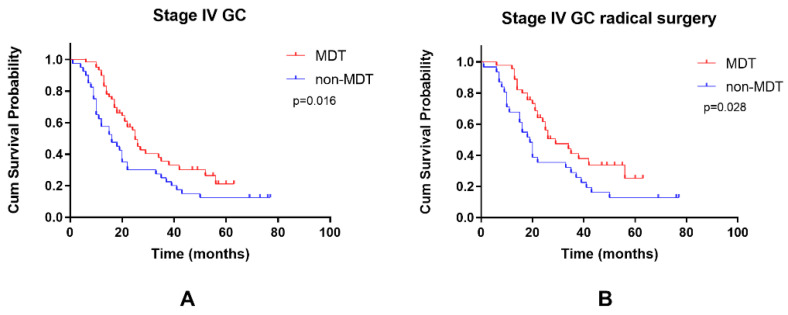
Kaplan-Meier curves of the OS of the two groups. (**A**) Stage IV GC (**B**) stage IV GC which underwent radical surgery.

Among stage IV GC patients, the 3-year OS rate of the MDT was 33.0%, and the 3-year OS rate of the non-MDT group was 25.0% (*p* = 0.016) (Figure 4A). The 3-year OS rate of MDT group patients with stage IV GC who underwent radical surgery was 41.1%, and that of the non-MDT group was 29.0% (*p* = 0.028) (Figure 4B). Multivariate analysis showed that MDT intervention and radical surgery were protective factors that reduced the mortality of stage IV GC (HR = 0.368, *p* = 0.001; HR = 0.323, *p* = 0.001), while N2 and N3 stages were adverse factors that increased the mortality of stage IV GC (Table 4).

## 4. Discussion

It is still controversial whether MDTs can improve the survival rate of advanced gastric cancer. We retrospectively analyzed the clinical data of advanced gastric cancer (GC) patients with and without MDT intervention, and compared the differences in their prognoses. No differences were found between MDT and non-MDT patients in general clinical information such as age, gender, tumor size, tumor location, degree of differentiation, Borrmann classification, tumor staging, CEA, or radical resection. Compared with proximal GC, stomach body cancer may be an unfavorable factor for the prognosis of stage III GC, possibly due to a higher metastasis rate of stomach body cancer to the peritoneum, liver, pancreas and other proximal sites. Another negative sign is elevated CEA which indicates tumor progression and, thus poorer prognosis; however, one must bear in mind that the level of CEA in stage IV GC is not necessarily higher. N3 is an unfavorable prognostic factor of stage III and IV GC; the later the N stage, the worst the bad biological behavior of the tumor and hence, a poorer outcome compared to the non-MDT group, The proportion of patients with stage T4 and N3 was significantly higher, indicating that the condition of the patients in the MDT group were more complicated, hence requiring more care and attention when diagnosing. Experience suggests that patients pre-diagnosed with T4 and N3 were deemed unresectable in most conventional cases; however upon closer inspection by various experts across the field, certain recent developments in experimental medication or chemotherapy regimens might be suggested to be beneficial and thus reduce the staging to operable levels. The survival curve indicated that MDT intervention can improve the overall survival rate of patients with advanced GC. Multivariate analysis revealed that MDT treatment and radical surgery were independent factors that improved the prognosis of patients with advanced GC. We further independently analyzed the effect of MDT intervention on the prognosis of patients with stage III and stage IV GC, and found that MDTs could improve their overall survival rate. Multivariate Cox regression analysis showed that MDT intervention was an independent influencing factor that improves the prognosis of stage III and stage IV GC.

Several studies have also shown that MDT evaluation can improve outcomes in patients with lung cancer [19], breast cancer [20], colorectal cancer [21], esophageal cancer [22] and prostate cancer [23]. These results further strengthen our final verdict, showing that MDTs can be effectively applied to many other complicated clinical cases, benefiting the lives of many.

There is a previous study that had shown that MDT intervention could increase the OS of early GC patients [5]. Another a retrospective cohort study in China showed that MDT could prolong the OS time of patients with metastatic esophageal carcinoma and gastroesophageal junction cancer [24]. At present, the impact of MDT intervention on the prognosis of advanced GC is still uncertain. Our research suggested that MDT intervention could decrease the mortality of advanced GC. It was deemed an independent protective factor that had reduced the mortality rate 0.507 times more than when no MDT intervention was provided. Bouvier et al. found that MDT discussion often increased the adjuvant therapy of GC patients undergoing radical resection [25]. As an important complement to the surgical treatment of locally advanced GC, adjuvant therapy can reduce tumor staging, increase the possibility of achieving radical surgery, prolong progression-free survival and overall survival, and improve prognosis [26]. For locally advanced GC, current guidelines recommend preoperative and postoperative radiotherapy and chemotherapy combined with radical gastrectomy, which can improve the long-term survival time of these advanced patients [27,28]. Du et al. found that MDT treatment could often revise the treatment plan, to allow for a more wholistic treatment and thus significantly improve the 5-year overall survival of gastrointestinal malignant tumors [29].

Stage IV GC is a difficult disease to treat even for the most specialized clinicians. In the past, it was thought that these patients had lost all opportunity for curative treatment and thus they were only given palliative care. Kinoshita et al. found that the prognosis of stage IV GC patients who underwent conversion therapy fared better than those who only received chemotherapy [30]. Another study pointed out that through MDT intervention, conversion therapy for patients with stage IV GC who responded well to chemotherapy can increase their OS [31]. Fukuchi et al. analyzed the clinical data of 151 patients with stage IV GC [32]. They found that the 40 patients who were successfully treated with conversion therapy lived longer than those of patients treated with just chemotherapy. Schildberg.et al. found that in 76 cases of metastatic GC and gastroesophageal junction cancer that were given palliative chemotherapy after MDT discussion, 16 successfully underwent conversion therapy. Eleven of them (69%) were able to achieve R0 resection, with four patients exceeding 60 months of survival time. For metastatic GC and gastroesophageal junction cancer patients that have reduced tumor size through chemotherapy, radical surgery after an MDT’s comprehensive evaluation could drastically improve the lifespan of patients [33]. In our study, stage IV GC was considered potentially resectable, and MDT intervention effectively prolonged the patients’ OS time. Therefore, for potentially resectable advanced GC and those with stage IV GC able to receive conversion therapy, MDT intervention can improve their prognosis, and thus it should be conducted throughout the treatment process.

However, this study had its limitations. First, our study was a retrospective study, and all patients were treated with gastrectomy, while inoperable patients were excluded, which may lead to selection bias. Second, disease-free survival was not fully recorded in the follow-up data and the impact of MDT intervention on disease-free survival in advanced GC could not be assessed. Third, although the two groups of patients were not treated and recorded in the same time frame, the surgical techniques in our center did not change during the study period, and there was no difference in the rate of radical surgery between the two groups. The chemotherapy regimens were selected according to the first-line chemotherapy regimens recommended by the NCCN guidelines of the time. Therefore, the effect of advancement in drug therapy on our results is acceptable. Finally, our study only included a small number of patients from a single center, which may not represent the general public, which is why more future research is warranted to portray a bigger picture.

## 5. Conclusions

Our study found that MDT had improved overall survival for advanced gastric cancer patients. We suggest that MDT discussion should be conducted for all advanced GC patients and a standardized methodology should be established. In addition, large, multicenter prospective studies are needed to further verify the effects of MDT on advanced gastric cancer.

## Figures and Tables

**Table 1:** Basic and Clinical characteristics of patients with advanced GC treated and not treated MDT intervention.

Characteristics	Non-MDT (*n* = 162)	MDT (*n* = 232)	*p*
Age (years)			
<60	93 (57.4)	143 (61.6)	0.399
≥60	69 (42.6)	89 (38.4)	
Sex			
Male	108 (66.7)	146 (62.9)	0.446
Female	54 (33.3)	86 (37.1)	
Primary tumor site			
Upper	47 (29.0)	85 (36.6)	0.284
Middle	41 (25.3)	62 (26.7)	
Lower	62 (38.3)	73 (31.5)	
Whole	12 (7.4)	12 (5.2)	
Radical resection			
No	23 (14.2)	37 (15.9)	0.634
Yes	139 (85.8)	195 (84.1)	
cT stage			
T1 + T2	4 (2.5)	5 (2.2)	0.000 ***
T3	106 (65.4)	90 (38.8)	
T4	52 (32.1)	137 (59.1)	
cN stage			
N0	5 (3.1)	32 (13.8)	0.000 ***
N1	23 (14.2)	30 (12.9)	
N2	84 (51.9)	65 (28.0)	
N3	50 (30.9)	105 (45.3)	
cTNM stage			
III	122 (75.3)	172 (74.1)	0.793
IV	40 (24.7)	60 (25.9)	
Differentiation			
Moderate	30 (18.5)	59 (25.4)	0.106
Poor	132 (81.5)	173 (74.6)	
Borrmann type			
I + II	25 (15.4)	41 (17.7)	0.558
III + IV	137 (84.6)	191 (82.3)	
CEA (ug/L)			
≤5	111 (68.5)	173 (74.6)	0.188
>5	51 (31.5)	59 (25.4)	

*** *p* < 0.001; MDT: multidisciplinary team; CEA: carcinoembryonic antigen.

**Table 2:** Univariate and multivariate analysis for OS in advanced GC.

Characteristics	Univariate Analysis			Multivariate Analysis		
	HR	95% CI	*p*	HR	95% CI	*p*
Age (years)						
<60	1.000			1.000		
≥60	1.056	0.813–1.371	0.684	1.204	0.912–1.590	0.191
Sex						
Male	1.000			1.000		
Female	1.138	0.872–1.475	0.341	1.156	0.868–1.540	0.320
Primary tumor site						
Upper	1.000			1.000		
Middle	0.920	0.648–1.305	0.639	0.623	0.425–0.912	0.015 *
Lower	1.035	0.756–1.417	0.829	0.828	0.595–1.153	0.263
Whole	2.039	1.243–3.346	0.005 **	1.192	0.706–2.010	0.511
Radical resection						
No	1.000			1.000		
Yes	0.147	0.106–0.203	0.000 ***	0.127	0.087–0.185	0.000 ***
cT stage						
T1 + T2	1.000			1.000		
T3	1.814	0.688–5.046	0.221	2.116	0.758–5.907	0.152
T4	1.616	0.595–4.393	0.346	2.433	0.862–6.866	0.093
cN stage						
N0	1.000			1.000		
N1	1.391	0.698–2.769	0.348	1.408	0.687–2.886	0.349
N2	1.645	0.894–3.025	0.110	1.883	0.959–3.698	0.066
N3	3.326	1.833–6.033	0.000 ***	3.760	1.938–7.295	0.000 ***
cTNM stage						
III	1.000			1.000		
IV	2.917	1.660–2.907	0.000	1.320	0.960–1.814	0.088
Differentiation						
Moderate	1.000			1.000		
Poor	1.976	1.379–2.832	0.000 ***	1.597	1.086–2.349	0.017 *
Borrmann type						
I + II	1.000			1.000		
III + IV	1.651	1.125–2.424	0.010 *	1.174	0.777–1.773	0.446
CEA (μg/L)						
≤5	1.000			1.000		
>5	1.360	1.032–1.791	0.029 *	1.420	1.058–1.907	0.020 *
Group						
Non-MDT	1.000			1.000		
MDT	0.689	0.528–0.899	0.006 **	0.493	0.365–0.667	0.000 ***

* *p* < 0.05, ** *p* < 0.01, *** *p* < 0.001; MDT: multidisciplinary team; HR: hazard ratio; CI: confidence interval; CEA: carcinoembryonic antigen.

**Table 3:** Univariate and multivariate analysis for OS stage III GC.

Characteristics	Univariate Analysis			Multivariate Analysis		
	HR	95% CI	*p*	HR	95% CI	*p*
Age (years)						
<60	1.000			1.000		
≥60	1.088	0.794–1.492	0.598	1.254	0.894–1.759	0.190
Sex						
Male	1.000			1.000		
Female	1.704	0.775–1.489	0.666	1.096	0.782–1.537	0.594
Primary tumor site						
Upper	1.000			1.000		
Middle	0.711	0.463–1.094	0.121	0.464	0.293–0.736	0.001 **
Lower	0.931	0.646–1.343	0.703	0.829	0.553–1.243	0.364
Whole	1.499	0.743–3.025	0.258	0.864	0.416–1.794	0.695
Radical resection						
No	1.000			1.000		
Yes	0.044	0.026–0.072	0.000 ***	0.044	0.025–0.077	0.000 ***
cT stage						
T1 + T2	1.000			1.000		
T3	1.309	0.414–4.138	0.646	1.97	0.581–6.676	0.276
T4	0.990	0.311–3.156	0.986	2.836	0.798–10.08	0.107
cN stage						
N0	1.000			1.000		
N1	1.145	0.514–2.55	0.74	0.721	0.317–1.639	0.435
N2	1.387	0.708–2.718	0.34	0.957	0.457–2.005	0.908
N3	3.173	1.635–6.16	0.001 **	2.203	1.038–4.677	0.040 *
Differentiation						
Moderate	1.000			1.000		
Poor	2.392	1.510–3.788	0.000 ***	2.206	1.336–3.643	0.002 **
Borrmann type						
I + II	1.000			1.000		
III + IV	1.542	1.005–2.365	0.047	1.426	0.895–2.271	0.135
CEA (μg/L)						
≤5	1.000			1.000		
>5	1.358	0.970–1.901	0.074	1.544	1.061–2.247	0.023 *
Group						
Non-MDT	1.000			1.000		
MDT	0.709	0.511–0.982	0.039 *	0.504	0.347–0.731	0.000 ***

* *p* < 0.05, ** *p* < 0.01, *** *p* < 0.001; MDT: multidisciplinary team; HR: hazard ratio; CI: confidence interval; CEA: carcinoembryonic antigen.

**Table 4:** Univariate and multivariate analysis for OS in stage IV GC.

Characteristics	Univariate Analysis			Multivariate Analysis		
	HR	95% CI	*p*	HR	95% CI	*p*
Age (years)						
<60	1.000			1.000		
≥60	1.113	0.695–1.781	0.656	1.413	0.834–2.393	0.198
Sex						
Male	1.000			1.000		
Female	1.240	0.781–1.969	0.361	1.048	0.585–1.878	0.874
Primary tumor site						
Upper	1.000			1.000		
Middle	1.452	0.752–2.804	0.267	1.476	0.665–3.275	0.338
Lower	1.137	0.601–2.150	0.693	1.173	0.584–2.354	0.654
Whole	2.202	1.001–4.844	0.050	1.920	0.783–4.713	0.154
Radical resection						
No	1.000			1.000		
Yes	0.529	0.315–0.888	0.016 *	0.323	0.167–0.624	0.001 **
cT stage						
T1 + T2	1.000			1.000		
T3	4.57	0.624–33.482	0.135	2.966	0.362–24.283	0.311
T4	4.628	0.632–33.869	0.131	4.051	0.479–34.256	0.199
cN stage						
N0	1.000			1.000		
N1	1.806	0.395–8.253	0.446	3.938	0.709–21.86	0.117
N2	4.119	0.952–17.816	0.058	11.408	2.189–59.466	0.004 **
N3	2.887	0.699–11.918	0.143	10.398	1.906–56.724	0.007 **
Differentiation						
Moderate	1.000			1.000		
Poor	1.219	0.680–2.185	0.505	1.314	0.699–2.469	0.397
Borrmann type						
I + II	1.000			1.000		
III + IV	1.389	0.729–2.645	0.317	1.300	0.576–2.931	0.528
CEA (μg/L)						
≤5	1.000			1.000		
>5	1.300	0.801–2.109	0.288	1.405	0.792–2.493	0.245
Group						
Non-MDT	1.000			1.000		
MDT	0.578	0.366–0.915	0.019 *	0.368	0.203–0.667	0.001 **

* *p* < 0.05, ** *p* < 0.01; MDT: multidisciplinary team; HR: hazard ratio; CI: confidence interval; CEA: carcinoembryonic antigen.

## Data Availability

The data presented in this study are available on request from the corresponding author.

## Supplementary Materials

The following supporting information can be downloaded at: https://www.mdpi.com/article/10.3390/curroncol29020102/s1, Table S1: Basic and Clinical characteristics of stage III GC patients treated and not treated by MDT intervention. Table S2: Basic and Clinical characteristics of stage IV GC patients treated and not treated MDT intervention.
