# Supplementary material for: The Prognostic Effect of Multidisciplinary Team Intervention in Patients with Advanced Gastric Cancer
Source: Curr Oncol. 2022 Feb 17;29(2):1201–12. doi: 10.3390/curroncol29020102 (PMC8871247; doi:10.3390/curroncol29020102)
Supplement: Supplementary file 1 [file curroncol-29-00102-s001.zip › curroncol-1567418-supplementary.pdf]

Table S1: Basic and Clinical characteristics of stage III GC patients treated and not treated by MDT intervention.

Characteristics	Non-MDT (n=122)	MDT (n=172)	*p*
**Age (years)**			
< 60	67 (54.9)	105 (61.0)	0.293
≥ 60	55 (45.1)	67 (39.0)	
**Sex**			
Men	84 (68.9)	109 (63.4)	0.330
Women	38 (31.1)	63 (36.6)	
**Primary tumor site**			
Upper	41 (33.6)	69 (40.1)	0.211
Middle	27 (22.1)	47 (27.3)	
Lower	49 (40.2)	49 (28.5)	
Whole	5 (4.1)	7 (4.1)	
**Radical resection**			
No	14 (11.5)	22 (12.8)	0.735
Yes	108 (88.5)	150 (87.2)	
**cT stage**			
T1 + T2	2 (1.6)	4 (2.3)	0.000***
T3	81 (66.4)	73 (42.4)	
T4	39 (32.0)	95 (55.2)	
**cN stage**			
N0	4 (3.3)	27 (15.7)	0.000***
N1	13 (10.7)	24 (14.0)	
N2	73 (59.8)	54 (31.4)	
N3	32 (26.2)	67 (39.0)	
**Differentiation**			
Moderate	23 (18.9)	46 (26.7)	0.116
Poor	99 (81.1)	126 (73.3)	
**Borrmann type**			
I + II	22 (18.0)	37 (21.5)	0.463
III + IV	100 (82.0)	135 (78.5)	
**CEA (ug/L)**			
≤ 5	84 (68.9)	131 (76.2)	0.164
> 5	38 (31.1)	41 (23.8)	

* $p<0.05$, ** $p<0.01$, *** $p<0.001$; MDT: multidisciplinary team; CEA: carcinoembryonic antigen

Table S2. Basic and Clinical characteristics of stage IV GC patients treated and not treated MDT intervention

Characteristics	Non-MDT (n=40)	MDT (n=60)	*p*
**Age (years)**			
< 60	26 (65.0)	38 (63.3)	0.865
≥ 60	14 (35.0)	22 (36.7)	

**Sex**			
Men	24 (60.0)	37 (61.7)	0.867
Women	16 (40.0)	23 (38.3)	
**Primary tumor site**			
Upper	6 (15.0)	16 (26.7)	0.236
Middle	14 (35.0)	15 (25.0)	
Lower	13 (32.5)	24 (40.0)	
Whole	7 (17.5)	5 (8.3)	
**Radical resection**			
No	9 (22.5)	15 (25.0)	0.774
Yes	31 (77.5)	45 (75.0)	
**cT stage**			
T1 + T2	2 (5.0)	1 (1.7)	0.001**
T3	25 (62.5)	17 (28.3)	
T4	13 (32.5)	42 (70.0)	
**cN stage**			
N0	1 (2.5)	5 (8.3)	0.484
N1	10 (25.0)	6 (10.0)	
N2	11 (27.5)	11 (18.3)	
N3	18 (45.0)	38 (63.3)	
**Differentiation**			
Moderate	7 (17.5)	13 (21.7)	0.610
Poor	33 (82.5)	47 (78.3)	
**Borrmann type**			
I + II	6 (15.0)	8 (13.3)	0.814
III + IV	34 (85.0)	52 (86.7)	
**CEA (ug/L)**			
≤5	27 (67.5)	42 (70.0)	0.828
>5	13 (32.5)	18 (30.0)	

* $p<0.05$, ** $p<0.01$, *** $p<0.001$; MDT: multidisciplinary team; CEA: carcinoembryonic antigen
